# Coronary Artery Bypass Grafting among Patients Undergoing Cardiac Surgery in a Tertiary Care Hospital: A Descriptive Cross-sectional Study

**DOI:** 10.31729/jnma.7233

**Published:** 2022-02-28

**Authors:** Praman Sharma, Lokesh Yadav, Rajesh Nepal, Sunil Babu Khanal, Subhadra Agrawal, Vivek Kattel

**Affiliations:** 1 Department of Cardiovascular and Thoracic Surgery, Nobel Medical College and Teaching Hospital, Biratnagar, Nepal; 2 Department of Obstetrics and Gynecology, BP Koirala Institute of Health Sciences, Dharan, Nepal; 3 Department of Internal Medicine, BP Koirala Institute of Health Sciences, Dharan, Nepal

**Keywords:** *cardiac surgical procedures*, *coronary artery bypass*, *heart failure*, *ventricular function*

## Abstract

**Introduction:**

Among all cardiac surgeries, coronary artery bypass graft is frequently performed and is expected to improve the quality of life in patients with coronary artery disease. This study aimed to look at the prevalence of the patients undergoing coronary artery bypass grafting among cardiac surgery cases at a tertiary care hospital.

**Methods:**

This was a descriptive cross-sectional study conducted at the Cardio-Thoracic and Vascular Surgery department of a tertiary care hospital among 92 patients who underwent cardiac surgery. Participants were enrolled after the ethical clearance from the Institutional Review Committee (Reference number: 574/2019). Epidemiological characters, functional New York Heart Association classification, and left ventricular function were evaluated. Data were entered in Microsoft Excel 2010 and analyzed using Statistical Package for the Social Sciences version 20. Point estimate at 95% Confidence Interval was calculated along with frequency and proportion for binary data.

**Results:**

The prevalence of coronary artery bypass graft in patients undergoing cardiac surgery was 49 (53.2%) (43.0-63.4 at 95% Confidence Interval). The mean age of 49 patients was 58.87 years. Hypertension 43 (88%), Dyslipidemia 34 (69%) and Type 2 Diabetes Mellitus 25 (51%) were major comorbidities. The mean preoperative and postoperative ejection fraction was 48.2±10.5% and 52.6±6.0% respectively. The preoperative median New York Heart Association class was 3 whereas the post-operative class was 1. Mortality was seen in one (2.04%) patient.

**Conclusions:**

The prevalence of coronary artery bypass graft in the patient undergoing cardiac surgery was lower than noted in other studies.

## INTRODUCTION

Among all cardiac surgeries, Coronary artery bypass graft (CABG) is frequently performed due to the high prevalence of coronary artery disease (CAD) worldwide.^1^ Quality of life is important as added by World Health Organization slogans "Add Years to Life" but also "Add life to years" in 1993.^2^

CABG has been tested for more than four decades to relieve symptoms and to reduce the risk of death in patients with CAD.^3,4^ Choice of conduits and the introduction of a variety of surgical techniques may influence patient outcomes.^5^ Survival outcome was U-shaped curve meaning the risk of mortality was high at early months of post CABG however with advancements in technology, equipment, skilled manpower, peri-operative care, and proper case selection there is a drastic decline in mortality.^6^

The aim of this study was to find out the prevalence of patients undergoing Coronary Artery Bypass Grafting in a tertiary care hospital.

## METHODS

This was a descriptive cross-sectional study conducted among 92 cases of cardiac surgeries in the Department of Cardiovascular and Thoracic Surgery of Nobel Medical College and Teaching Hospital. Ethical approval was taken from the Institutional Review Committee (Reference number: 574/2019). These patients were enrolled from June 2019 to March 2021 after taking written informed consent. All cases were followed up for at least a 12 weeks period. The convenience sampling technique was used. The sample size was estimated as,

n = Z^2^ × p × q / e^2^

  = (1.96)^2^ × 0.7 × (1-0.7) / (0.01)^2^

  = 81

Where,

n = minimum required sample size
Z = 1.96 at 95% Confidence Interval (CI)
p = prevalence of CABG surgery in a cardiac center taken from a previous study, 70%^7^
q = 1-p
e = margin of error, 10%

Accounting for 10% non-response rate, the total number of participants needed for the study was 90. We included 92 participants. The variables measured were baseline clinical characteristics, comorbidities, New York Heart Association (NYHA) class, Left Ventricular Ejection Fraction (LVEF), coronary angiography findings, perioperative complications, and duration of hospital stay. LVEF was measured subjectively by visual estimation and objectively re-confirmed by M mode or modified Simpson method.

Data were entered in Microsoft Excel 2010 and analyzed using IBM Statistical Package for the Social Sciences version 20. Continuous data are reported as mean and standard deviation, categorical data as frequency and percentage. Pre and post-left ventricle functions were presented with reference to NYHA and LVEF. Point estimate at 95% CI was calculated.

## RESULTS

Out of the total 92 cardiac surgeries, 49 (53.2%) (43.063.4 at 95% Confidence Interval) were coronary artery bypass grafting. Among 49 CABG cases, there was one (2%) in-hospital mortality whereas the remaining 48 (98%) were successfully followed up for three months. The mean and median age of the CABG patients were 58.87 and 58.0 years respectively. Forty-five (92%) of the CAD were triple vessel disease (TVD) followed by three (6%) Double Vessel Disease (DVD) and one (2%) Single Vessel Disease (SVD). Four (8%) had left main stem disease. Hypertension, dyslipidemia, diabetes, and thyroid disease were present among 43 (88%), 34 (69%), 25 (51%), and eight (16%) respectively (Table 1).

**Table 1:** Baseline epidemiological characteristics (n = 49).

Epidemiological characteristics	n (%)
**Gender**	
Female	25(51)
Male	24 (49)
**Underlying comorbidity**	
Hypertension	43 (87.8)
Dyslipidemia	34 (69.3)
Diabetes	25(51)
Hypothyroidism	7(14.3)
Hyperthyroidism	1 (2.0)
Arrhythmia	3(6.1)
Valvular heart disease	2 (4.0)
Congenital cardiac anomalies	1 (2.0)
**Associated risk factor**	
Smoking and tobacco consumption	13 (26.5)
Sedentary lifestyle	12 (24.5)
Family history of cardiac disease	9(18.3)
Family history of cardiac surgery	2(4.1)
Previous history of PTCA	2(4.1)

Perioperative inotropes and blood transfusion were used among 46 (93.8%) and 39 (79.6%) cases respectively. One (2%) patient went under IABP during the CABG procedure. The mean pump time was 88 minutes with a range of 56-192 minutes. The mean aortic cross-clamp time was 52 minutes with a range of 27-149 minutes. The mean ventilator support duration was 6 hours with a range of 4-12 hours. Median ICU stay was 2 days whereas eight (16.3%) patients had single day ICU stay and one (2%) patient had 5 days stay. The median hospital stay was 6 days whereas one (2%) patient was discharged after 3 days and two (4%) patients were admitted for 12 days before discharge. Nine (18.3%) patients had postoperative arrhythmia. Postoperative acute kidney injury (AKI) was present among seven (14.2%) cases two (4%) required hemodialysis (Table 2).

**Table 2:** Perioperative characteristics (n = 49).

Perioperative variables	n (%)
**Use of inotropes**	
None	3(6.1)
Single	11 (22.4)
Double	27(55.1)
Triple	8(16.3)
**Blood transfusion**	
None	10(20.4)
One pint	8(16.7)
Two pints	13(26.5)
Three pints	11 (22.4)
>3 pints	7 (14.2)
**Pump duration (minutes)**	
<60	2 (4.1)
61-90	27 (55.1)
91-120	12 (24.4)
120-240	8 (16.3)
**Cross clamp duration (minutes)**	
<30	1 (2.0)
31-45	11 (22.4)
46-60	23 (46.9)
61-90	13 (26.5)
91-120	1 (2.0)
**ICU stay (days)**	
1-3	47 (95.9)
4-6	2 (4.0)
**Hospital stay (days)**	
1-5	14 (28.5)
6-10	32 (65.3)
>10	3 (6.1)
**Others**	
IABP	1 (2.0)
Hemodialysis	2 (4.0)
Re surgery	2 (4.0)
Mortality	1 (2.0)

**Figure 1:**
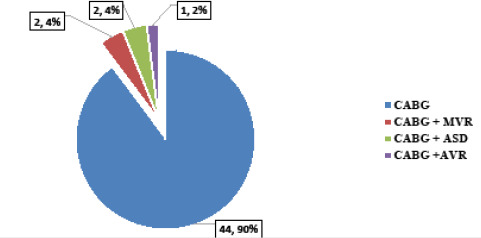
Types of surgeries (n= 49).

One (2%) patient had to consider re-surgery for sternum dehiscence. Patients with other cardiac diseases were also repaired (Figure 1).

Preoperative mean and median EF were 48.2±10.5% and 50% respectively. Postoperatively the mean and median EF was 52.6±6% and 55% respectively. Preoperatively median NYHA class was 3 whereas the postoperative median NYHA class was 1 respectively (Table 3).

**Table 3:** Preoperative and 90-days postoperative values of LVEF and NYHA functional class.

	Preoperative (n= 49) n (%)	90 days postoperative (n= 48) n (%)
**LVEF**		
<30%	6 (12.2)	-
31-45%	15 (30.6)	9 (18.7)
>45%	28 (57.1)	39 (81.2)
**NYHA class**		
Class 1	-	34 (70.8)
Class 2	1 (2)	14 (29.2)
Class 3	27 (55.1)	-
Class 4	21 (42.8)	-

## DISCUSSION

In this descriptive cross-sectional study, out of 92 consecutive cases of cardiac surgeries 49 patients underwent CABG. The prevalence of CABG in the patients undergoing cardiac surgery was 53.2%, which was lower than noted in other studies.^7^ This could be due to the increased frequency of Rheumatic Heart Disease in our population and their subsequent surgeries.^8^ The mean and median age in our study was less than 60 years whereas in the developed country it is much higher.^6^ Prevalence of CAD in early ages in our setup as compared to developed countries could have been due to genetic predisposition for coronary heart disease risk factors such as possibly the metabolic syndrome in South Asia and increased exposure to risk factors. The male gender was more common to have CABG. Globally there is a tendency for female patients to receive less aggressive invasive and pharmacological treatment after ACS. However, in our study male and female ratio are almost equal (49:51). This difference in our study could have been due to the small sample size.^9^

Risk factors for CAD like hypertension, dyslipidemia, and diabetes were present among more than 50% of the cases. The INTERHEART study showed that there were potentially modifiable risk factors for ACS including raised Apo B/Apo A ratio, current smoking status, psychosocial factors, diabetes, hypertension, abdominal obesity, alcohol consumption, regular physical activity, and daily consumption of fruits and vegetables.^10^

CABG has been considered as independent of the perioperative factors associated with the improvement of quality of life.^6^ In CAD patients with LV dysfunction, there is marked improvement in LV functions and it may even normalize in some patients which leads to improvement in functional NYHA class shown by different studies. Preoperatively NYHA class 2, 3, and 4 symptoms were present among one (2%), 27 (55.1%), and 21 (42.8%) respectively. After 12 weeks NYHA class 1 and 2 status was among 34 (70.8%) and 14 (29.2%) patients respectively. Preoperative ejection fraction <30%, 31-45% and >45% were six (12.2%), 15 (30.6%) and 28 (57.1%) respectively. Postoperative ejection fraction 31-45% and >45% were nine (18.7%) and 39 (81.2%) respectively. This significant improvement in the outcome was comparable to the studies done by Caputti GM, et al, Islamoglu F, et al. and Mickleborough LL, et al.^11-13^ Various factors are responsible for the outcome such as preoperative LV systolic dysfunction severity, lesion pattern, case selection, surgical hands, complete revascularization, proper myocardial protection, cardiac anesthesia management, emergency cardiac facilities, and postoperative intensive care monitoring and management.^14,15^ To determine the outcome in decreased LVEF, a study was conducted by the Canadian Cardiovascular Society, which revealed a 42% improvement in Angina Class, thus concluded CABG can be performed safely for improving LVEF and quality of life.

Chertow, et al. in their study show renal injury/ failure in cardiac surgery varies between 5-30% and needing hemodialysis is 1-5%, which is comparable to our study. The percentage of the patients developing postoperative complications in the form of AKI needing hemodialysis in our study was 4% which was comparable. Factors like perioperative renal ischemia or reduced renal reserve lead to postoperative renal insufficiency; increase in creatinine of more than 0.5 mg/dl or a relative increase of more than 25% from the baseline has been commonly used in different studies. The proper method of preventing this type of complication and treatment strategies are urgently needed.^16^

Post cardiac surgery bleeding is well known serious complication, requiring reexploration, blood transfusion, increased in-hospital stay, and cost leading to increase morbidity and mortality.^17^ In our study, 4% of patients underwent reexploration for excessive bleeding which is a bit higher than other studies. An increased tendency of bleeding was observed in high body mass index, high European System for Cardiac Operative Risk Evaluation (EURO) score, urgent/ emergency surgeries, increased creatinine level, and low platelet counts of patients. Re-exploration leads to significant blood transfusion, adverse effects on cardiorespiratory functions, increased period of mechanical ventilation support, prolonged intensive care unit stay, hospital stay, and increase mortality.^18^

Perioperative mortality in our study was 2% as compared to the Danish cohort of 51307 CABG patients 3.2%.^5^ The differences could be due to small sample size, differences in population age group, and variation of comorbidities between two population groups. 30-day mortality risk after CABG surgery decreased from 3.1% from 1999 to 2000 to 2.4% during 2011 to 2012, while the 1-year risk remained unchanged.^19^

The limitations of study was small sample size in a single tertiary center. Participants were enrolled in sequential manner rather than randomization. We used left Internal mammary artery (LIMA) and saphenous vein grafts only and did not use right internal mammary artery (RIMA) or radial grafts. We did not study the myocardial viability imaging in patients with low LV function which could have an impact on outcome.

## CONCLUSIONS

The prevalence of CABG in the patients undergoing cardiac surgery was lower than noted in other studies. This could be due to the increased frequency of rheumatic heart disease in our population and their subsequent surgeries. The baseline characteristics of the participants undergoing CABG were similar to other studies. The mortality was also comparable.
